# Risk factors for wound healing complications after revascularization for MMD with complete Y-shaped incision

**DOI:** 10.1038/s41598-022-18709-4

**Published:** 2023-02-24

**Authors:** Chenchao Wang, Hongwei Li, Yang Dong, Hao Wang, Dongpeng Li, Chengbin Zhao, Lei Cao, Kaiwen Sun, Jiefeng Geng, Bo Yang

**Affiliations:** Department of Neurosurgery, The First Affiliated Hospital of Zhengzhou University, No. 1, Jianshe East Road, Erqi District, Zhengzhou, 450053 Henan China

**Keywords:** Diseases, Medical research

## Abstract

Moyamoya disease (MMD) is a chronic occlusive cerebrovascular disease that can be treated with revascularization. Surgery increases the risk of poor wound healing (PWH) due to the impact on the blood supply to the flap. We aimed to analyze risk factors for PWH in MMD with a complete Y-shaped incision. A total of 125 patients with MMD were enrolled in this prospective observational study. The wounds were assessed and measured on the third and seventh days after surgery. The mean age of these patients was 43.3 ± 10.0 years. The ratio of male to female was 1:1.3. 15 (12.0%) patients had incision complications. 5 patients (4.0%) had redness; 2 patients (1.6%) had swelling; 2 patients (1.6%) had fat necrosis; 3 patients (2.4%) had incision infection; and 3 patients (2.4%) had flap necrosis. Student’s t test showed significant differences in BMI (*P* = 0.040) and fever time (*P* = 0.050). The standard chi-squared test showed significant differences in incision infection (*P* = 0.010), suture mode (*P* = 0.047), and cutting off large branch vessels in the flap (*P* < 0.001). Multivariate logistic regression analysis suggested that incision infection (*P* = 0.026, *OR* 12.958), using a skin stapler (*P* = 0.030, *OR* 4.335), cutting off large branch vessels in the flap (*P* = 0.009, *OR* 5.227), and BMI (*P* = 0.027, *OR* 1.204) were risk factors. The area under the curve for risk factors for PWH on a receiver operating characteristic curve was 0.853. Incision infection, using a skin stapler, higher BMI, and cutting off large branch vessels in the flap are risk factors for PWH.

## Introduction

Moyamoya disease (MMD) is a chronic and progressive steno-occlusive cerebrovascular disease^1^. The MMD prevalence peaks at two ages: around 10 years and at 30–45 years^2^. Ischemic events are the most common initial presentation while hemorrhagic strokes are more frequent in adults^3^. It can be treated by surgical revascularization^4^. Many studies have verified the effectiveness of extracranial-to-intracranial (EC-IC) bypass surgery to prevent cerebral ischemia in MMD^4–6^. There are three main types of bypass surgery, including indirect, direct, and combined revascularization. Indirect revascularization mainly includes encephaloduroarteriosynangiosis (EDAS), encephalomyosynangiosis (EMS) and encephaloduroarteriomyosynangiosis (EDAMS). Direct revascularization mainly refers to the superficial temporal artery to middle cerebral artery (STA-MCA) anastomosis. Combined revascularization includes STA-MCA bypass + EDAS, STA-MCA bypass + EMS and STA-MCA bypass + EDAMS.

As the most effective and common EC-IC bypass surgery, direct and combined bypass always choose Y-shaped incision for more convenient vascular separation and smaller flaps. However, these procedures impair the blood supply to the scalp by providing the STA to the brain. It always results in poor wound healing (PWH), causing incision pain, swelling, scalp necrosis, skull exposure, CSF leakage, and intracranial infection.

At present, there is little evidence about the risk factors for PWH of MMD. Takanari et al. thought using both branches of the STA and history of diabetes mellitus rather than age and gender were risk factors for MMD^7^. Acker et al. considered PWH incidence depended on the revascularization strategy and skin incision applied, with a complete Y-shaped incision giving the worst results after analyzing the wound healing process in the patients who underwent a direct or combined bypass surgery with a focus on different skin incisions^8^. To reduce the incidence of PWH of Y-shaped incision, we used Logistic regression analysis to identify risk factors for PWH in patients with Y-shaped incision.

## Materials and methods

This prospective cohort study collected patients from the Department of Neurosurgery of the First Affiliated Hospital of Zhengzhou University. The study was performed in accordance with the Declaration of Helsinki, relevant guidelines and regulations.All participants signed informed consent forms approved by the local ethics committee. A total of 149 patients with MMD were admitted to our department from March 2021 to December 2021. 125 patients were enrolled in this study. Their clinical data were collected, including age, gender, hypertension, diabetes, hyperlipidemia, incision characteristics, suture mode, and healing status.

Patients with MMD were included in the study if surgical revascularization was conducted with a complete Y-shaped incision, and no previous scars or signs of infection. Patients were excluded from study if they did not receive revascularization surgery, the surgical incision was not complete Y-shaped, vital information was missing or patients were lost to follow up.

### Definition of PWH

The criteria for PWH were as follows: (1) The incision was swollen and painful. (2) The incision exuded for more than 3 days. (3) The skin of the incision became dark and hard. (4) The margin was necrotic and ulcerated. (5) Unhealed incision or incision dehiscence occurred 7 days after the operation. (6) Purulent exudate was present from the wound.

### Incisional procedure

Before the operation, the main STA, frontal branch and parietal branch of the STA were marked on the temporal skin. The first skin incision is made along the frontal branch, then the second skin incision is made along the parietal branch ending in the first incision. Finally, form a complete Y or V-shaped incision. All operations are performed by four surgeons with equal surgical skills in one team.

## Statistical methods^9^

Numerical variables were analyzed by using Student’s t test or Mann–Whitney U test. Categorical variables were analyzed by using the chi-squared test or Fisher’s exact test. Multivariate logistic regression was conducted to evaluate the potential risk factors associated with PWH. Receiver operating characteristic curves (ROCs) were established, and the area under the ROC curve (AUC) was determined to evaluate the predictive quality of PWH. The statistical analyses were conducted using IBM SPSS version 26 (IBM Crop., Armonk, NY, USA). P-value < 0.05 was considered statistically significant.

### Ethical approval and consent to participate

Written consent was obtained from all research individuals or guardians. The medical ethics committee of the First Affiliated Hospital of Zhengzhou University approved the study plan.

## Results

### Demographic characteristics of the overall study population

A total of 125 patients with MMD were identified. The mean age of these patients was 43.3 ± 10.0 years. The ratio of male to female was 1:1.3. 15 (12.0%) patients had wound healing complications. Of these patients, 5 patients (4.0%) had redness along the edge of the incision; 2 patients (1.6%) suffered from flap swelling; 2 patients (1.6%) had fat necrosis; 3 patients (2.4%) suffered from incision infection; 3 patients (2.4%) went through flap necrosis and underwent restoration surgery (Table 1).

**Table 1:** The type of poor wound healing.

Type	Number	Proportion (%)
Incision redness	5	4.0
Flap swell	2	1.6
Fat necrosis	2	1.6
Incision infection	3	2.4
Incision dehiscence	0	0
Flap necrosis	3	2.4
Total	15	12.0

### Comparison of characteristics between the poor wound-healing group and the good wound-healing group

The patients were divided into two groups according to wound healing. After normality testing, all numerical variables except albumin were included in Student’s t test. There were significant differences in BMI (*P* = 0.040) and duration of fever (*P* = 0.050) between patients in the poorly healed and well healed groups (Table 2). The results of the Mann–Whitney U test showed that the difference in albumin between the two groups was not statistically significant (*P* = 0.879). Significant differences in incisional healing existed after stratifying patients by incisional infection (*P* = 0.010), suture pattern (*P* = 0.047), and large branch vascular cut of the flap (*P* < 0.001), and cardinal analysis was performed (Table 3).

**Table 2:** Comparison of quantitative factors.

Factor	Poor wound-healing group	Good wound-healing group	*T*	*P*
	$\overline{{\text{x}}}$ ± s	$\overline{{\text{x}}}$ ± s		
Age (year)	44.94 ± 6.78	43.04 ± 10.40	0.708	0.480
BMI	27.57 ± 3.98	25.71 ± 3.99	2.08	0.040
Operation time (min)	161.25 ± 29.24	146.91 ± 28.95	1.848	0.067
Fever time (day)	4.00 ± 2.45	2.83 ± 2.15	2.000	0.050
Flap base length (cm)	7.52 ± 0.67	7.22 ± 1.33	1.406	0.168
Flap height (cm)	4.69 ± 0.94	4.54 ± 1.00	0.589	0.557
Incision length (cm)	11.38 ± 1.75	11.95 ± 1.58	1.718	0.088
Base length/height	1.70 ± 0.43	1.66 ± 0.46	0.296	0.768
Incision angle (°)	75.07 ± 17.91	80.70 ± 15.41	− 1.337	0.184

**Table 3:** Comparison of qualitative factors.

Factor		Poor wound-healing group	Good wound-healing group	*χ*^2^	*P*
		16 (12.8%)	109 (87.2%)		
Gender	Male	9 (56.3%)	45 (41.3%)	1.273	0.259
	Female	7 (43.8%)	64 (58.7%)		
Diabetes	No	14 (87.5%)	96 (88.1%)	< 0.001	1.000*
	Yes	2 (12.5%)	13 (11.9%)		
Hyperlipidemia	No	10 (62.5%)	72 (66.1%)	0.078	0.780
	Yes	6 (37.5%)	37 (33.9%)		
Hypertriglyceridemia	No	10 (62.5%)	88 (80.7%)	2.739	0.098
	Yes	6 (37.5%)	21 (19.3%)		
Hypercholesterolemia	No	8 (66.7%)	90 (79.6%)	0.449	0.503*
	Yes	4 (33.3%)	23 (20.4%)		
Low HDL lipoproteinemia	No	14 (87.5%)	105 (96.3%)	0.840	0.359*
	Yes	2 (12.5%)	4 (3.7%)		
High LDL lipoproteinemia	No	10 (62.5%)	78 (71.6%)	0.550	0.459
	Yes	6 (37.5%)	31 (28.4%)		
Hypertension	No	11 (68.8%)	71 (65.1%)	0.081	0.776
	Yes	5 (31.3%)	38 (34.9%)		
Use of STA	Single	15 (93.8%)	105 (96.3%)	< 0.001	1.000*
	Both	1 (6.3%)	4 (3.7%)		
Incision infection	No	13 (81.3%)	107 (98.2%)	4.705	0.010*
	Yes	3 (18.8%)	2 (1.8%)		
Suture mode	Intradermal suture	10 (62.5%)	91 (83.5%)	3.961	0.047
	Using skin stapler	6 (37.5%)	18 (16.5%)		
Cutting off large branch vessels in the flap	No	6 (37.5%)	88 (80.7%)	13.984	< 0.001
	Yes	10 (62.5%)	21 (19.3%)		

*Continuity correction.

### Multivariable logistic regression analysis of poor wound healing

A multivariable logistic regression analysis model was used to research the risk factors for PWH after EC-IC bypass surgery for MMD with a complete Y-shaped incision after the above comparative analyses. Multivariable logistic regression analysis showed that incision infection (*P* = 0.026, OR 12.958, 95% CI 1.367–122.872), using a skin stapler (*P* = 0.030, *OR* 4.335, 95% CI 1.156–16.257), cutting off large branch vessels in the flap (*P* = 0.009, *OR* 5.227, 95% CI 1.156–16.257) and BMI (*P* = 0.027, *OR* 1.204, 95% CI 1.021–1.418) were independent risk factors for poor wound healing (Table 4).

**Table 4:** Binomial multivariate logistic regression analysis of poor incision healing.

Factor	*B*	*S.E*	*Wald*	*P*	*OR*	95% CI of *OR*	
						Lower	Upper
Incision infection	2.562	1.148	4.729	0.026	12.958	1.367	122.842
Stapling skin	1.467	0.674	4.729	0.030	4.335	1.156	16.257
BMI	0.185	0.084	4.891	0.027	1.204	1.021	1.418
Cutting off large branch vessels in the flap	1.663	0.636	6.837	0.009	5.277	1.517	18.358
Constant	− 8.176	2.476	10.907	0.001			

### Evaluation of the predictive quality of PWH

**Figure 1:**
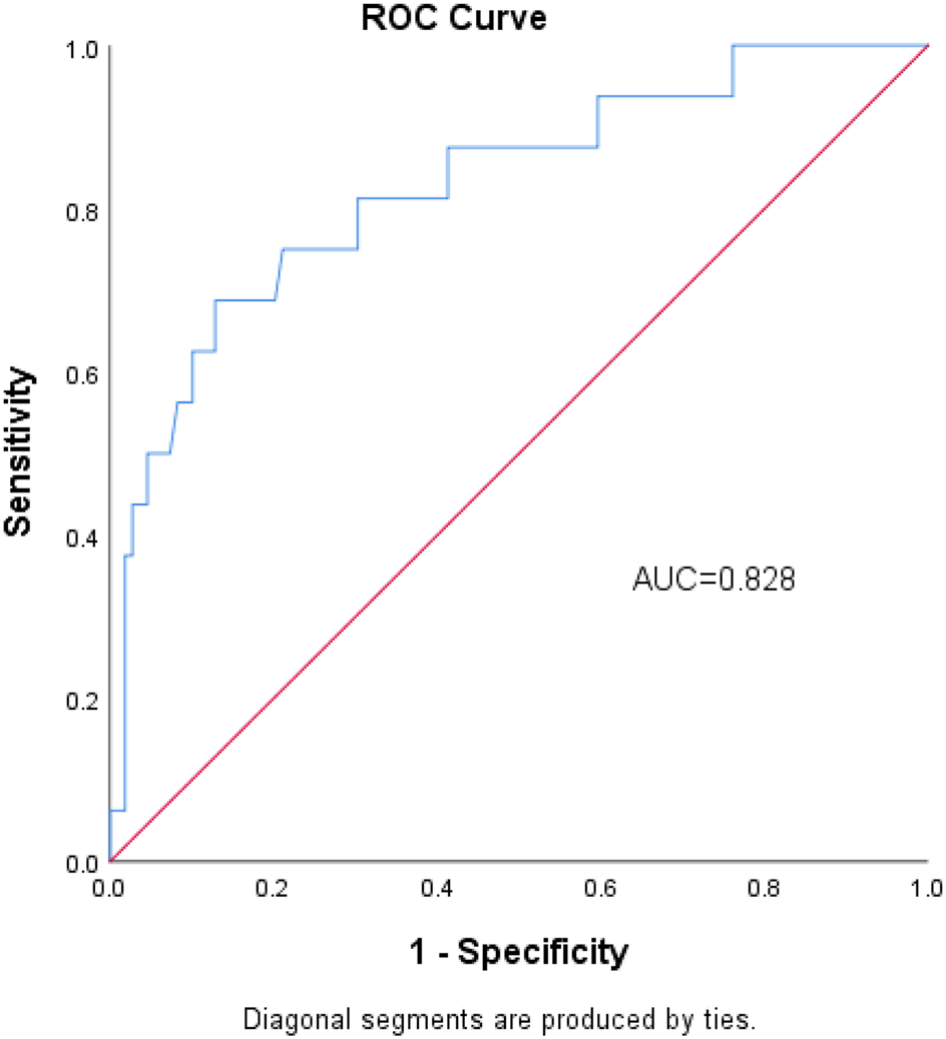
ROC curve of predictive model.

The multivariate logistic regression model fit the data well with the Hosmer–Lemeshow goodness-of-fit test (P = 0.980). The ROC curve of predicted probability was constructed based on the multivariate logistic regression model (Fig. 1). The predicted AUC of the wound healing complication model after revascularization was 0.828 (95% CI 0.712–0.944). Therefore, the predictive model performs well in predicting wound healing complications after complete Y-shaped incision revascularization.

## Discussion

Revascularization easily causes PWH because providing extra blood vessels for bypass surgery aggravate the insufficiency of blood supply of the flap in MMD^10^. A total of 7.7–21.4% of the patients who underwent cerebral revascularization surgery developed wound-related complications^7,10–12^. Acker et al. observed that using complete Y-shaped incision had the highest wound complication rate, accounting for 19%^8^. In our research, the incidence of PWH was 12.0%, slightly lower than the incidence of PWH in the previous study. In our current research, incision infection, using a skin stapler, higher BMI, and cutting of large branch vessels in the flap can be good indicators to predict PWH. Furthermore, these risk factors can be changed to avoid PWH and have practical significance.

Our research showed that incision infection was the leading risk factor. It was the same as the previous study. Shanmugam et al. believed that wound infection was an important factor for PWH^13,14^. In a systematic review, the mean operative time was approximately 30 min longer in patients with surgical site infection compared with those patients without^15^. Our mean operative time was 148.74 ± 29.27 min, and cefuroxime was given preoperatively to prevent infection. Although there was no statistical difference in the operative time between the two groups, the mean operative time of the poor wound healing group was 161.25 ± 29.24 min, longer than that of the control group (146.91 ± 28.95 min), and closer to the time limit of 180 min for re-supplementation of antibiotics. Therefore, we think that a longer operative time may increase the risk of infection. The wound healing process usually becomes complicated due to infection^15^. Individual species may alter virulence and quantity as well as the formation of a biofilm, which further impedes the efficacy of the host response and thus delays repair^16^. Infection can also cause fever and inflammatory responses. Subsequently, the body enters a state of negative nitrogen balance. It is not conducive to the healing of the wound. It is noteworthy that a small proportion of our male patients were diagnosed with seborrheic dermatitis by dermatologists before surgery. Such a scalp environment would provide a proper habitat for microbial growth. We suggest that the patient take measures to control the scalp environment before the operation. For doctors, changing the dressing more frequently and applying antibacterial ointments after the operation are effective methods.

Using skin stapler is another risk factor for PWH in our research. Suture helps to disperse the tension across the incision^17^ and promotes the healing of the incision. However, there is no comparative study on skin suture methods in craniotomy. Smith et al. thought that there was a significantly higher risk of developing a wound infection when the wound is closed with staples rather than sutures after orthopaedic surgery^18^. But his view caused a lot of controvers^18–20^. Although using skin stapler is quick and convenient, we prefer continuous intradermal sutures nowadays. In our research, the wound complication rate of using skin stapler (25%) was much higher than continuous intradermal suture (9.9%). Nevertheless, an error caused by the small sample size should be considered. The Reason for skin stapler causing PWH is not apparent. It could be due to the staples being too thick for flap corner, leading to skin ischemia. Although the continuous intradermal suture is complex, suture with 4-0 or 5-0 synthetic absorbable suture material has the advantage that there is no need for suture removal. In addition, barbed sutures of cosmetic suturing have transformed the way surgeons approximate wounds by eliminating knots, distributing wound tension, and increasing the efficiency of closure^21^. In general, the continuous intradermal suture is better than using skin stapler.

In addition, high BMI is a significant risk factor. Lanzetti et al. did a Logistic regression analysis of 90 patients with surgically treated ankle fracture and identified that higher BMI could delay the wound healing^22^. The cause of PWH is related to obesity^23^. Obesity can impair the healing response and result in a chronic nonhealing wound^24^. Patients with higher BMI have thicker subcutaneous fat, which can easily cause fat liquefaction, obstructing incision healing. Hirt et al. thought increased BMI affected skin physiology, the skin barrier and increased the incidence of bacterial and Candida skin infections^25^. A higher BMI dulls the body’s response to infection and impairs wound healing. Therefore, dressings should be changed frequently when treating patients with higher BMI. At the same time, PWH could be discovered and treated in time. Avishai et al. thought that too low BMI may predispose an individual to delayed and even impaired wound healing because malnutrition could not meet the nutritional need for cell proliferation and protein synthesis^26^. But in our study, 3.2% of patients had a BMI ≤ 18.5and there were no patients with BMI ≤ 18.5 in the poor wound healing group. We cannot prove that low BMI is a risk factor for PWH. More researches are needed to confirm the impact of low BMI on PWH of MMD.

**Figure 2:**
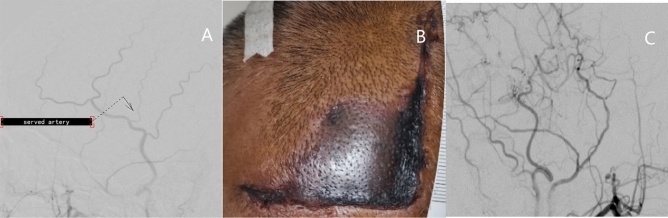
This group of pictures shows the temporal skin arteries and skin necrosis of a 57-year-old MMD male patient before and after the operation. (**A**) The preoperative DSA showed that there was a thick artery branch of Frontal branch of the left STA in the flap, which was cut during the bypass surgery. (**B**) Flap necrosis occurred in the postoperative patient. (**C**) 6 months after the flap restoring surgery, the peripheral artery compensated for the blood supply of the flap.

Last but not least, cutting off large branch vessels in the flap is a vital risk factor. Although 5 vessels supplying the cutaneous, subcutaneous, and galea aponeurosis running in the subcutaneous layer arteries form a network^27^, the STA supplies main area of the temporal region^28^. Kwon et al. thought insufficiency of scalp circulation due to excessive hemostasis from using electrocautery during the procedure and STA used for arterial anastomosiscause scalp necrosis^29^. Severing the branch of the STA decreases the blood supply of the temporal skin obviously. Takanari et al. believed that a trend toward more severe complications was demonstrated for the procedures that used both STA branches in comparison with the procedures that used only 1 STA branch^7,30^. In their study, it was STA-MCA bypass (× 2) procedure and the STA-MCA bypass + EDAMS procedure, not STA-MCA bypass + EDAMS procedure, EDAMS (× 2) procedure or EDAS (× 2) procedure that caused wound complication, which suggests that severing the main branches of STA for arterial anastomosis is an important risk factor for PWH. The reason why our results differ from theirs may be due to the using different procedures and statistical errors. In addition, we proved that cutting of large branch vessels, the secondary branch of the STA in the flap, was a risk factor for PWH. We believe that we have more accurately found the culprit vessel for severe PWH such as flap necrosis. Although we agree with Takanari’s view, it could not explain why the flap always showed necrosis first, instead of necrosis on both sides of the incision at the same time. We think that the large secondary branch is the main blood vessel to support the flap. Conversely, flaps that do not have a large secondary branch may have other sources of blood supply from the base. We found that patients with severe flap necrosis had at least 1 serve large secondary branch of the STA (Fig. 2). We think cutting these large arterial branches leads to ischemic necrosis in the flap. Therefore, preserving the blood supply vessel of the flap is the key to avoiding wound complications. Yoshimura et al. thought double STA-MCA bypass via a small craniotomy might be less invasive, especially for patients at high risk for postoperative cutaneous necrosis^31^. Some studies believed that the incidence of complications of incomplete Y-shaped incisions or linear incisions is lower than that of complete Y incisions^8,32^, and changing the craniotomy plan is an excellent choice to avoid damage to the blood supply of the skin. Kubo et al. recommended separating the blood vessels on the galea aponeurosis under the endoscope^30^ or using the in-to-out (ITO) dissection method^33^, which could be safely performed with the achievement of a less invasive dissection of the STA and an overall improved cosmetic outcome^34^.

Similar to previous studies, we proved that age, gender, hypertension, and hyperlipidemia are not risk factors for PWH^7^. However, we are surprised that diabetes, a widely recognized risk factor for PWH^7,22^, is not a risk factor. This may be an error caused by the small sample size. Interestingly, our study shows that most angles of complete Y-shaped incisions (range 50°–114°) will not cause PWH. However, we still need to avoid creating narrow flaps when designing the flaps.

Nevertheless, our study has some limitations. The most significant limitation is the relatively small sample size, and it is a single-center study. Moreover, the low number of events may also result in type I error, where some of the variables identified as significant may have not been truly associated with PWH. In addition, this study didn’t include factors such as atherosclerosis, smoking, alcohol abuse and the use of an electrotomy to cut skin. Finally, this study is only observational research and further studies are required to explore the molecular mechanisms of our findings.

## Conclusion

Incision infection, using a skin stapler, higher BMI, and cutting off large branch vessels in the flap are risk factors for wound healing complications after revascularization for MMD with a complete Y-shaped incision.

## Data Availability

The datasets generated during and/or analysed during the current study are available from the corresponding author on reasonable request.

## Abbreviations

MMD: Moyamoya disease
PWH: Poor wound healing
BMI: Body mass index
STA: Superficial temporal artery
MCA: Middle cerebral artery
ROC: Receiver operating characteristic
AUC: Area under the curve
CI: Confidence interval
OR: Odds ratio
CSF: Cerebrospinal fluid
EDAMS: Encephalduro-arterio-myo-synangiosis
EDAS: Encephalo-duro-arterio-synangiosis
